# HIV-1 Myristoylated Nef Treatment of Murine Microglial Cells Activates Inducible Nitric Oxide Synthase, NO_2_ Production and Neurotoxic Activity

**DOI:** 10.1371/journal.pone.0130189

**Published:** 2015-06-11

**Authors:** Giorgio Mangino, Marylinda Famiglietti, Caterina Capone, Caterina Veroni, Zulema Antonia Percario, Stefano Leone, Gianna Fiorucci, Sebastian Lülf, Giovanna Romeo, Cristina Agresti, Tiziana Persichini, Matthias Geyer, Elisabetta Affabris

**Affiliations:** 1 Department of Science, University Roma Tre, Rome, Italy; 2 Department of Medico-Surgical Sciences and Biotechnologies, Sapienza University of Rome, Latina, Italy; 3 Department of Cell Biology and Neuroscience, Istituto Superiore di Sanità, Rome, Italy; 4 Institute of Molecular Biology and Pathology, CNR, Rome, Italy; 5 Center of Advanced European Studies and Research, Group of Physical Biochemistry, Bonn, Germany; Ghent University, BELGIUM

## Abstract

**Background:**

The potential role of the human immunodeficiency virus-1 (HIV-1) accessory protein Nef in the pathogenesis of neuroAIDS is still poorly understood. Nef is a molecular adapter that influences several cellular signal transduction events and membrane trafficking. In human macrophages, Nef expression induces the production of extracellular factors (*e*.*g*. pro-inflammatory chemokines and cytokines) and the recruitment of T cells, thus favoring their infection and its own transfer to uninfected cells via exosomes, cellular protrusions or cell-to-cell contacts. Murine cells are normally not permissive for HIV-1 but, in transgenic mice, Nef is a major disease determinant. Both in human and murine macrophages, myristoylated Nef (myr^+^Nef) treatment has been shown to activate NF-κB, MAP kinases and interferon responsive factor 3 (IRF-3), thereby inducing tyrosine phosphorylation of signal transducers and activator of transcription (STAT)-1, STAT-2 and STAT-3 through the production of proinflammatory factors.

**Methodology/Principal Findings:**

We report that treatment of BV-2 murine microglial cells with myr^+^Nef leads to STAT-1, -2 and -3 tyrosine phosphorylation and upregulates the expression of inducible nitric oxide synthase (iNOS) with production of nitric oxide. We provide evidence that extracellular Nef regulates iNOS expression through NF-κB activation and, at least in part, interferon-β (IFNβ) release that acts in concert with Nef. All of these effects require both myristoylation and a highly conserved acidic cluster in the viral protein. Finally, we report that Nef induces the release of neurotoxic factors in the supernatants of microglial cells.

**Conclusions:**

These results suggest a potential role of extracellular Nef in promoting neuronal injury in the murine model. They also indicate a possible interplay between Nef and host factors in the pathogenesis of neuroAIDS through the production of reactive nitrogen species in microglial cells.

## Introduction

The term “neuroAIDS” encompasses different clinical syndromes including sensory neuropathy, myelopathy, HIV-associated dementia (HAD), HIV-associated encephalitis (HIVE) and cognitive/motor disorders. Despite their heterogeneity, these neurologic disorders are all characterized by neuronal loss, due to damage of central and peripheral nervous system by HIV (for review see [1]). Prior to highly active antiretroviral therapy (HAART) introduction, neurological disorders were the first manifestation of symptomatic HIV-1 infection, affecting roughly 10/20% of patients and up to 60% of patients in the advanced stages of HIV-1/AIDS [2]. HAART has reduced the incidence of severe forms of AIDS-associated neurologic disorders such as HIV-1-associated dementia (HAD) but, with longer life span, the prevalence of milder forms of neurologic manifestations such as HIV-1-associated neurocognitive disorder (HAND) appears to be increasing [3, 4].

Although substantial advances in deciphering the pathophysiological role of viral products in the development of neuroAIDS have been achieved, two main aspects of HIV-associated neuronal degeneration remain puzzling. First, neurological diseases are generally a later manifestation of HIV-1 infection and tend to progress in parallel with the degree of immunosuppression, while invasion of the nervous system may occur early upon viral infection, probably concomitant with initial systemic infection [5]. Second, since the virus is unable to infect neurons, the neuronal loss associated with neuroAIDS manifestations is not thought to be a direct cytopathic effect of HIV-1 itself [6, 7]. Accumulating evidence suggests that over-activation of the immune system, particularly of the macrophage subset, is the pathophysiological event common to all of the neuroAIDS-associated syndromes [8]. Macrophage activation produces pro-inflammatory cytokines, platelet activating factor (PAF), nitric oxide, and free oxygen radicals that appear to contribute to the neuronal damage and dysfunction underlying the clinical syndromes [7, 9–11]. Thus, neuroAIDS shares some similarities with other neurodegenerative syndromes such as Alzheimer’s disease, Parkinson's disease and multiple sclerosis, in which activated macrophages and microglial cells (*i*.*e*. the nervous system’s resident macrophages) act as crucial disease determinants by sustaining an inflammatory microenvironment that ultimately leads to nervous system damage.

Besides the production of inflammatory cytokines (interleukin, IL-1β, IL-6 and tumor necrosis factor-α, TNFα) the macrophages and microglia inflammatory response is characterized by the induction of oxygen and nitrogen reactive species (ROS and RNS, respectively), the latter following the activation of type 2 nitric oxide synthase (NOS2 or iNOS). In phagocytic cells, inflammatory stimuli such as bacterial lipopolysaccharides (LPS), coupled to the production of IFNγ by activated Th1 cells, trigger the NF-κB pathway and the expression of interferon responsive factor 1 (IRF-1) [12]. These two transcription factors cooperate by binding their specific target sequences located in the *inos* promoter region, thus inducing the synthesis of the enzyme [13–15]. In contrast to neuronal and endothelial NOS (nNOS and eNOS, respectively), iNOS is constitutively active once synthesized and acts in a Ca2^+^-independent manner [16, 17].

HIV-1 Nef is a small (MW 27–34 kDa) myristoylated, cytoplasmic, multifunctional virulence factor acting as an adaptor molecule inside the cell. It is partially associated with the cell membrane and plays multiple roles during HIV-1 replication [18–20]. Nef-defective viruses lead to an attenuated clinical phenotype with reduced viral load in mouse models, monkeys, and humans [21–25]. More recently, it has been shown that this viral protein can be transferred to uninfected cells via cellular nanotubes, cell-to-cell contacts and release of exosomes. These findings lead to the idea that Nef is able to regulate both the endocytotic and exocytotic cell pathways thereby inducing specific effects also in non-infected cells [26].

In human monocyte-derived macrophages (MDMs), both Nef expression within the cell and cell treatment with the recombinant protein induce a pro-inflammatory response characterized by synthesis and release of specific cytokines and chemokines [27–32]. Nef-induced pro-inflammatory state in macrophages is largely due to NF-κB activation [28, 32–34]. In addition, we reported that Nef treatment of MDMs activates IRF-3, the main transcriptional regulator leading to the synthesis of IFNβ [32] and, ultimately, to the induction of IRF-1. Based on these two premises, we hypothesized that Nef promotes synthesis and activation of iNOS in microglial cells as a result of its pro-inflammatory properties. Consequently, iNOS-derived nitrogen reactive species might play a role in neuronal loss in a Nef-dependent manner. Due to the lack of an available *in vitro* system based on human-derived microglial cells, we resorted to a well characterized murine microglial cell line (*i*.*e*. BV-2, see [35]) and tested the above hypotheses by treating cells with myristoylated (myr^+^) Nef and by analyzing iNOS and RNS induction, as well as the ability of RNS to induce neuronal death. Murine macrophages respond to Nef treatment as human MDMs by activating IKKα and IKKβ, JNK, and p38 MAP kinases [36]. Activation of the NF-κB pathway is mandatory for the tyrosine phosphorylation of signal transducer and activator of transcription (STAT)-1, STAT-2, and STAT-3, which is induced within 2 h in an autocrine and paracrine manner. These data confirmed that murine and human macrophages respond similarly to myr^+^Nef treatment [36].

Although murine cells are not permissive for HIV-1 infection, several investigators have engineered transgenic (Tg) mice to model HIV-1-induced diseases and overcome such restriction [37–39]. The generation of Tg mice expressing selected HIV-1 genes revealed that Nef represents a major disease determinant and that the murine system is a suitable model to investigate the mechanisms of Nef activity [37]. Nevertheless, in these studies the functions affected by Nef in monocytes/macrophages have not been extensively investigated. To the best of our knowledge, only two studies dealt with Nef expression in the brain of Tg mice: in the first one Nef was expressed in oligodendrocytes [40], whereas in the second Nef was expressed in macrophages/microglial cells but pro-inflammatory responses were not investigated [41].

In the present study, we describe that Nef treatment of murine microglial cells induces iNOS expression that requires both NF-κB activation and *ex novo* synthesis of IRF-1, an event dependent on IFNβ release. We also show that, similarly to other proinflammatory stimuli, such as LPS, extracellular Nef cooperates with IFNβ to induce iNOS. The myristoylation site and the acidic cluster of the viral protein are required for these effects. Finally, one or more factor(s), released in the supernatants of Nef-treated BV-2 microglial cells, induce neuronal death in a Nω-Nitro-L-arginine methyl ester (L-NAME) sensitive way.

## Results

### Extracellular Nef induces STATs phosphorylation, Iκ-B degradation and IRF-1 expression in BV-2 microglial cells

**Fig 1:**
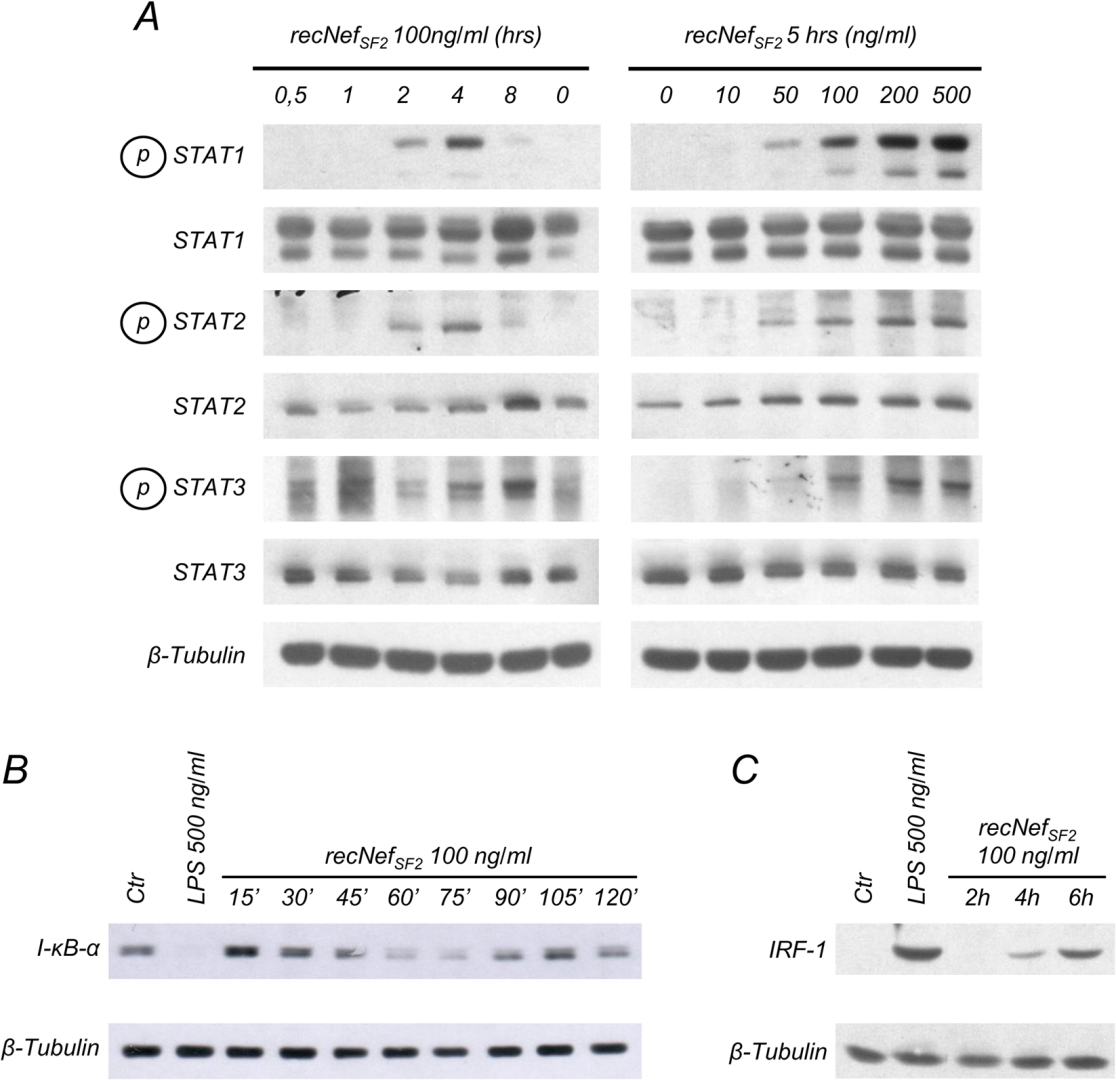
Nef treatment induces STATs tyrosine phosphorylation, I-B degradation and IRF-1 expression in BV-2 microglial cells. (**A**) BV-2 cells were left untreated or incubated with 100 ng/ml wild type myristoylated Nef derived from HIV-1 SF2 strain (myr^+^Nef_SF2_) for different times (left panel) or for 5 h with different amounts of myr^+^Nef_SF2_ (right panel). Total cellular extracts were analyzed by Western Blot to evaluate STAT-1, -2 and -3 tyrosine phosphorylation or protein expression using specific antibodies as described in materials and methods. (**B**, **C)** Cells were incubated for the indicated time with myr^+^Nef_SF2_ (100 ng/ml) or, as a positive control, with LPS (500 ng/ml) for 30’ in (**B**) and 6 h in (**C**). Whole cell lysates were analyzed by Western Blot for I-κB-α (**B**) or IRF-1 (**C**) expression. β-tubulin expression was used as an internal loading control. Results reported in the figure are from four independent experiments.

Two main transcription factors are responsible for iNOS/NOS2 induction in murine as well as human phagocytic cells, *i*.*e*. IRF-1 and NF-κB. Studies of the iNOS murine promoter revealed the presence of two NF-κB and one IRF-1 binding sites [13–15]. We previously reported that Nef activates the NF-κB signaling pathway, and synthesis of IRF-1 both in primary human MDMs and murine macrophages [28–30, 32, 36]. Therefore, we assessed whether this was also the case in microglial cells. First, we confirmed that myr^+^Nef_SF2_ treatment induced tyrosine phosphorylation of STAT-1, -2 and -3 in BV-2 cells in a time- and dose-dependent manner. In particular, tyrosine phosphorylation signal increased starting from 2 h of cell treatment with 100 ng/ml of the viral protein and was clearly detectable after 5 h of treatment with 50 ng/ml myr^+^Nef_SF2_ (Fig 1A).

Subsequently, cells were challenged for different times (from 15 to 120 min) with myr^+^ Nef_SF2_ and total cellular lysates were analyzed for the expression of I-κB-α, the alpha isoform of the inhibitor of κB. As shown in Fig 1B, myr^+^Nef_SF2_ treatment induced I-κB-α degradation that became clearly evident after 60 to 75 minutes. At later time points, protein levels started to recover, likely due to the neo-synthesis of I-κB which is itself encoded by a NF-κB regulated gene [42, 43]. After 4/6 h of myr^+^Nef_SF2_ treatment the expression of IRF-1, encoded by a STAT-1 responsive gene, was clearly increased (Fig 1C). These results indicate that in murine microglial cells Nef positively affects the two transcriptional factors involved in iNOS regulation.

### Nef treatment of murine microglial cells induces iNOS expression and NO_2_^-^ production

**Fig 2:**
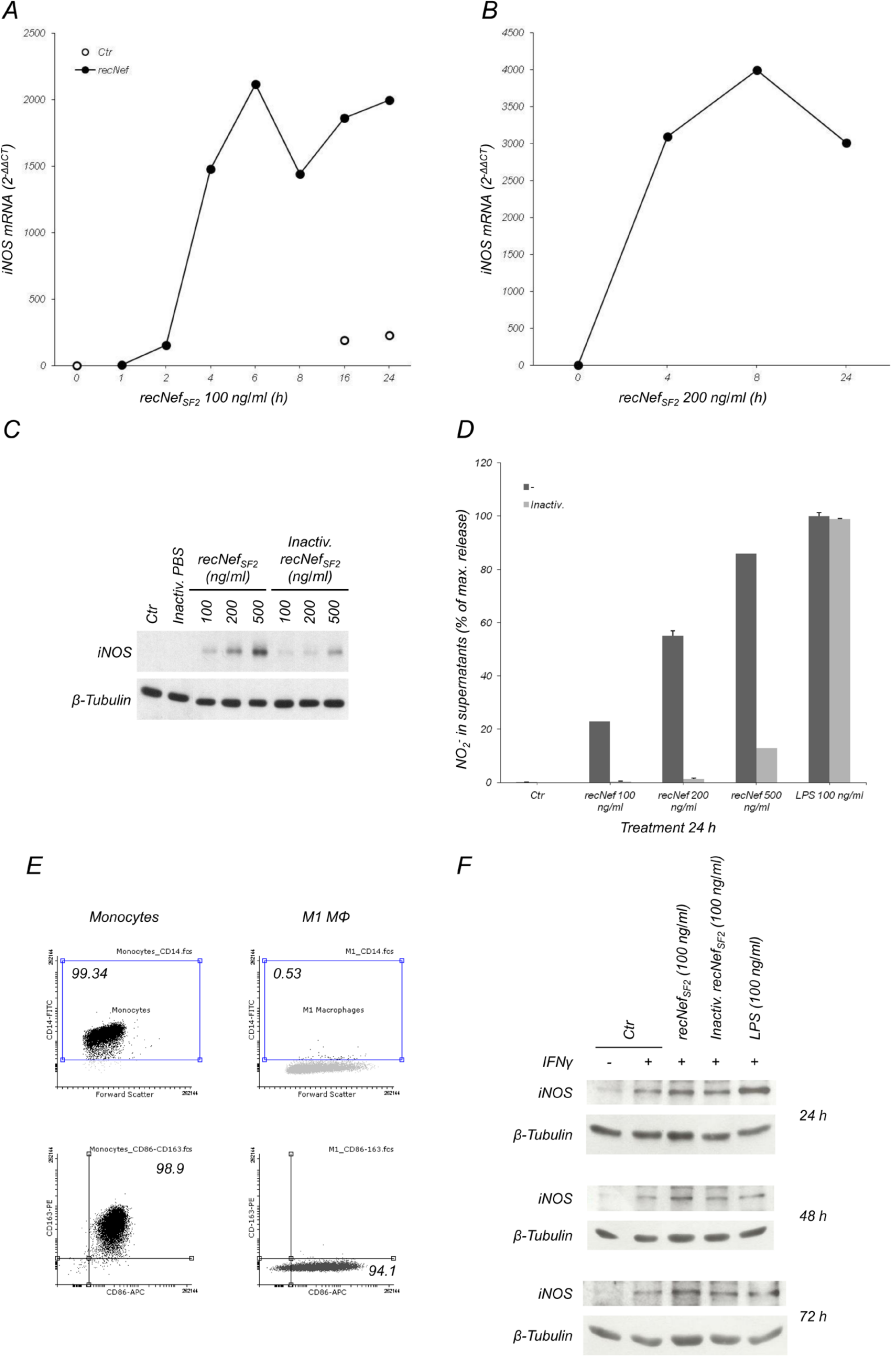
Nef treatment of microglial cells induces iNOS expression. BV-2 cells (**A**) or purified primary murine microglial cells (**B**) were treated for the indicated time with myr^+^Nef_SF2_ (100 ng/ml in **A**, 200 ng/ml in **B**, closed circles). Total cellular RNAs were isolated and real time RT-PCR analysis was performed as reported in the materials and methods section. Results were expressed using the 2^-ΔΔCT^ method using basal mRNA level in untreated cells (Ctr, open circles) at T = 0 as a calibrator and GAPDH level as an internal loading control. (**C**, **D**) BV-2 cells were treated for 24 h with the indicated amounts of myr^+^Nef_SF2_, heat inactivated myr^+^Nef_SF2_ (Inactiv. recNef), LPS or heat treated LPS. Total cell lysates were analyzed by Western Blot for iNOS (**C**, upper panel) levels and, as internal loading control, β-Tubulin expression (**C**, lower panel). (**D**) NO_2_^-^ content in the supernatants was quantified using the Griess colorimetric assay as reported in the materials and methods section. Dark gray bars: native myr^+^Nef or LPS. Light gray bars: heat pre-treated (Inactiv.) myr^+^Nef or heat pre-treated LPS. Results from one of five independent experiments are shown. (**E**) Cell phenotyping by flow cytometry of human monocytes and M1 macrophages obtained as described in materials and methods. According to [77], human monocytes were CD14^+^/CD163^+^/CD86^+^ whereas M1 macrophages were CD14^-^/CD163^-^/CD86^bright^. (**F**) M1 human macrophages were left untreated or treated for 24, 48 and 72 h with IFNγ, 100 ng/ml wild type myr^+^Nef_SF2_ plus IFNγ, heat pre-treated myr^+^Nef_SF2_ and IFNγ or 100 ng/ml LPS plus IFNγ. Total cellular extracts were analyzed by Western Blot to evaluate iNOS expression using specific antibodies as described in materials and methods. β-tubulin expression as internal loading control. Blots are representative of two independent experiments.

Based on the above results, we asked whether extracellular Nef was able to regulate iNOS expression. First, BV-2 cells were exposed to myr^+^Nef_SF2_ and iNOS mRNA levels were evaluated by Real Time RT-PCR from 1 to 24 h post-treatment. As shown in Fig 2A, iNOS mRNA expression reached a peak at 6 h post-treatment, declined thereafter and increased again at 16 and 24 h, similarly to the kinetics of iNOS mRNA induction in cytokines-stimulated DLD-1 cells [44]. Induction of iNOS mRNA expression was also confirmed in primary murine microglial cells (Fig 2B). To test whether iNOS expression and NO_2_^-^ production were both affected, BV-2 cells were treated for 24 h with 100 to 500 ng/ml myr^+^Nef_SF2_. iNOS protein levels were measured in total cellular extract, whereas quantification of NO_2_^-^ released into the supernatants was performed using the Griess reagent. As depicted in Fig 2C and 2D, iNOS expression and NO_2_^-^ release were induced in a dose-dependent manner by myr^+^Nef treatment of microglial cells. The effect required the integrity of the protein, as heat-denaturated Nef barely induced iNOS synthesis and NO_2_^-^ release. As a positive control, we evaluated NO_2_^-^ production induced by LPS which is not sensitive to heat inactivation. To verify iNOS induction in a human context, we polarized monocytes into M1 inflammatory macrophages by cultivating them for 6 days in the presence of GM-CSF (Fig 2E). M1 macrophages were then treated with myr^+^Nef in the presence of IFNγ for 24, 48 or 72 h. We were unable to detect nitrite accumulation in the supernatants of Nef-treated M1 macrophages, due to the well known low NO_2_^-^/NO_3_^-^ ratio production in human cells [45], nevertheless we found that iNOS was slightly induced in cells treated for 48 h with myr^+^Nef plus IFNγ compared to IFNγ treatment alone (Fig 2F and S1 File). Stimulation with LPS plus IFNγ, used as a positive control, induced iNOS at 24 and 48 h declining thereafter at 72 h (Fig 2F).

### iNOS upregulation induced by extracellular Nef requires both NF-κB and IFNβ

**Fig 3:**
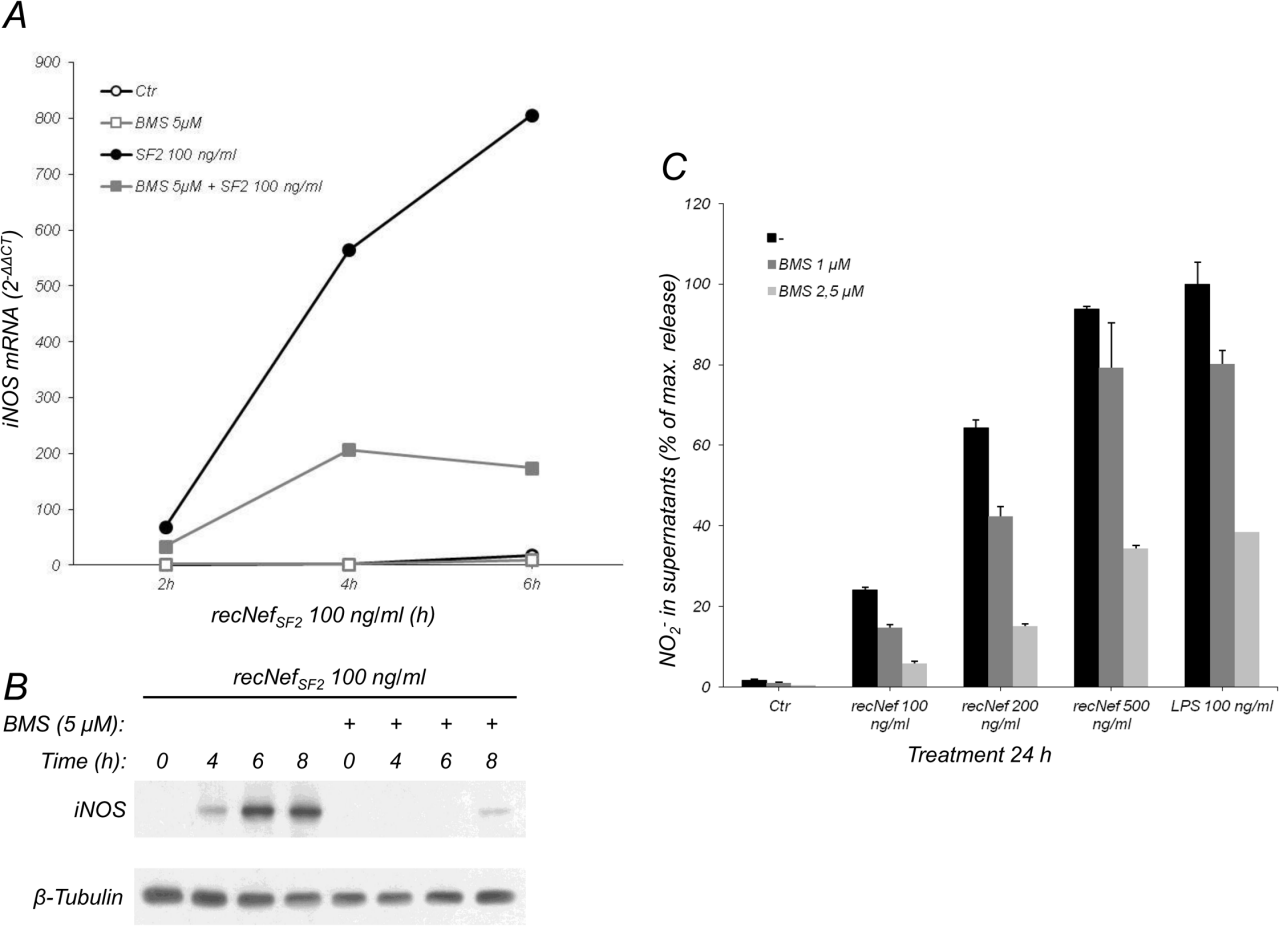
Nef-dependent iNOS induction requires NF-κB. (**A**) BV-2 cells were pretreated for 1 h with BMS-345541 (5 μM) and then incubated for 2, 4 and 6 h with myr^+^Nef_SF2_ (100 ng/ml) in presence of BMS-345541. Total mRNAs were isolated and analyzed for iNOS mRNA expression as described in the materials and methods section. Empty circles: untreated cells (Ctr); gray empty squares: BMS-345541; filled circles: recNef_SF2_; gray filled squares: BMS-345541 plus recNef_SF2_. (**B**) Cells were stimulated as in (**A**) for 4, 6 and 8 h, then lysed and total cell lysates analyzed by Western Blot for iNOS expression. β-Tubulin was used as an internal loading control. (**C**) BV-2 cells were pre-treated with BMS-345541 (1 or 2.5 μM), then stimulated for 24 h with the indicated amount of myristoylated recNef_SF2_ in presence of BMS-345541. Supernatants were collected and NO_2_^-^ content evaluated. Black bars: no inhibitor; dark gray bars: BMS-345541 at 1 μM concentration; light gray bars: BMS-345541 at 2.5 μM concentration. Ctr: control cells. Data shown were from one of three independent experiments.

As previously reported, iNOS expression is regulated by both NF-κB and IRF-1. To test whether the NF-κB pathway was involved in Nef-dependent iNOS induction, BV-2 cells were treated with recombinant myr^+^Nef_SF2_ in the presence of BMS-345541, a highly specific IKKα/IKKβ inhibitor. Pre-treatment with BMS-345541 at 5 μM concentration greatly reduced Nef-dependent induction of iNOS mRNA levels (Fig 3A) and completely inhibited iNOS expression (Fig 3B). However, due to BMS-345541 cytotoxicity upon prolonged cell treatment, NO_2_^-^ production was evaluated in supernatant of BV-2 cells treated with Nef for 24 h in the presence of lower doses of the inhibitor (*i*.*e*. 1 and 2.5 μM). Nonetheless, these doses were still able to reduce NO_2_^-^ production in a dose-dependent manner (Fig 3C).

**Fig 4:**
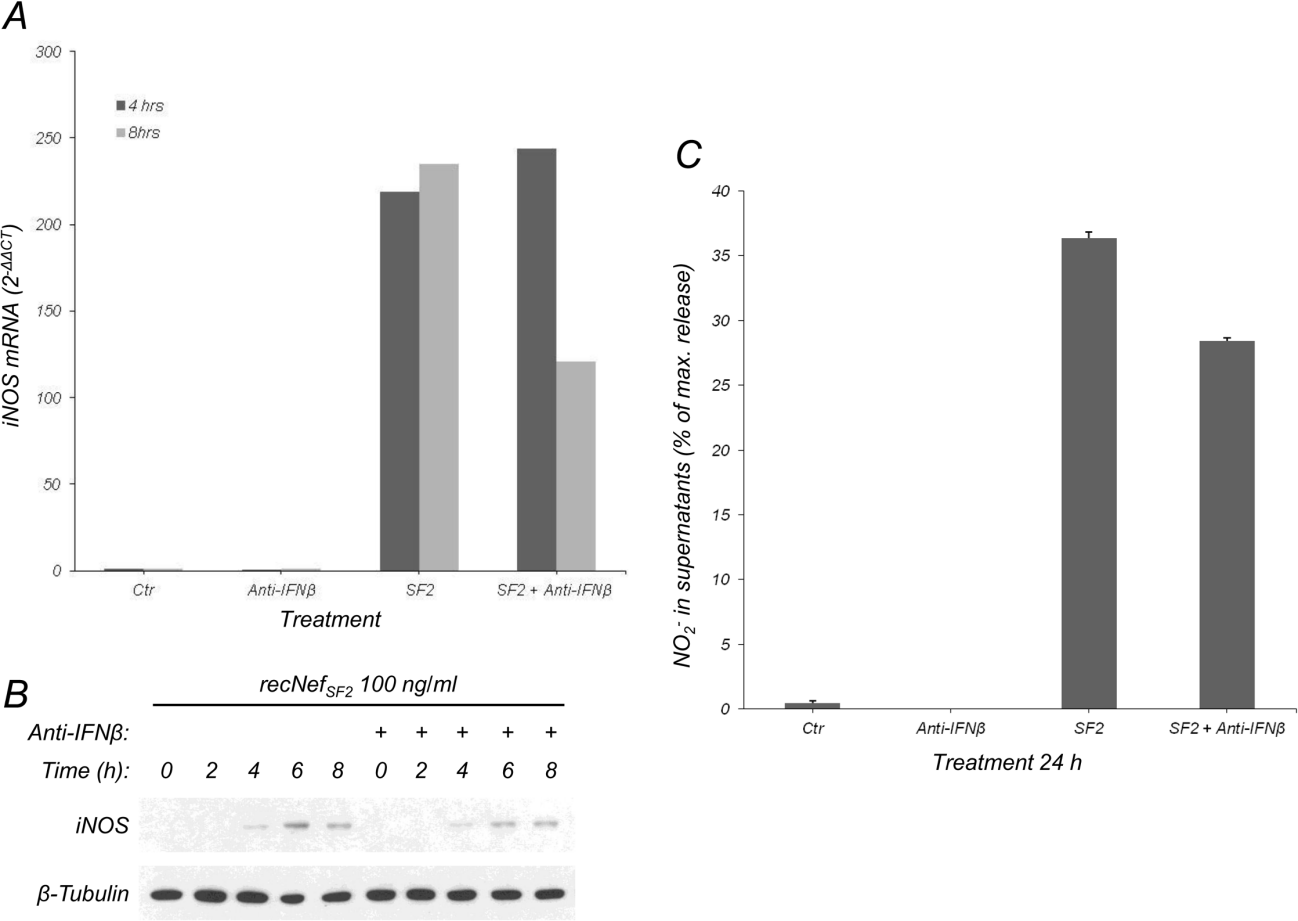
Nef-induced IFNβ production concurs to Nef-dependent iNOS induction. (**A**) BV-2 cells were treated for the indicated time with myr^+^Nef_SF2_ (100 ng/ml) in the presence of anti-IFNβ neutralizing antibodies as described [36]. iNOS mRNA was analyzed as reported in materials and methods section. Dark gray bars: 4 h treatment; light gray bars: 8 h treatment. (**B**) Cells were treated as in (**A**) for the indicated time and iNOS expression evaluated by Western Blot. β-Tubulin expression was used as an internal loading control. (**C**) Cells were treated as in (**A**) for 24 h, supernatants were then collected and NO_2_^-^ content measured using the Griess colorimetric assay.

In human MDMs we observed that Nef treatment induces both the synthesis and the release of IFNβ followed by IRF-1 production [30, 32]. We also reported that Nef treatment induced IRF-1 in RAW264.7, a murine macrophage cell line [36] and in BV-2 microglial cells (Fig 1C). Based on these findings, we hypothesized that IFNβ blockade could affect iNOS upregulation. BV-2 cells were incubated with myr^+^Nef_SF2_ in presence of anti-IFNβ neutralizing antibodies (NAb). As shown in Fig 4, anti-IFNβ NAb started to inhibit the induction of iNOS mRNA at 8 h post-treatment (Fig 4A). This is in agreement with the kinetics of IRF-1 protein induction, that follows Nef-induced degradation of Iκ-B (see Fig 1B). Consequently, iNOS expression was also affected by anti-IFNβ NAb starting from 6 h (Fig 4B, compare signals at 6 h in anti-IFNβ treated and untreated cells). NO_2_^-^ detected in the culture supernatants appeared also reduced after 24 h of treatment (Fig 4C). Taken together, these results demonstrated that Nef-induced iNOS upregulation is primarily dependent on NF-κB signalling and, to a lesser extent, on IFNβ production.

### Extracellular Nef cooperates with IFNβ to induce iNOS expression

**Fig 5:**
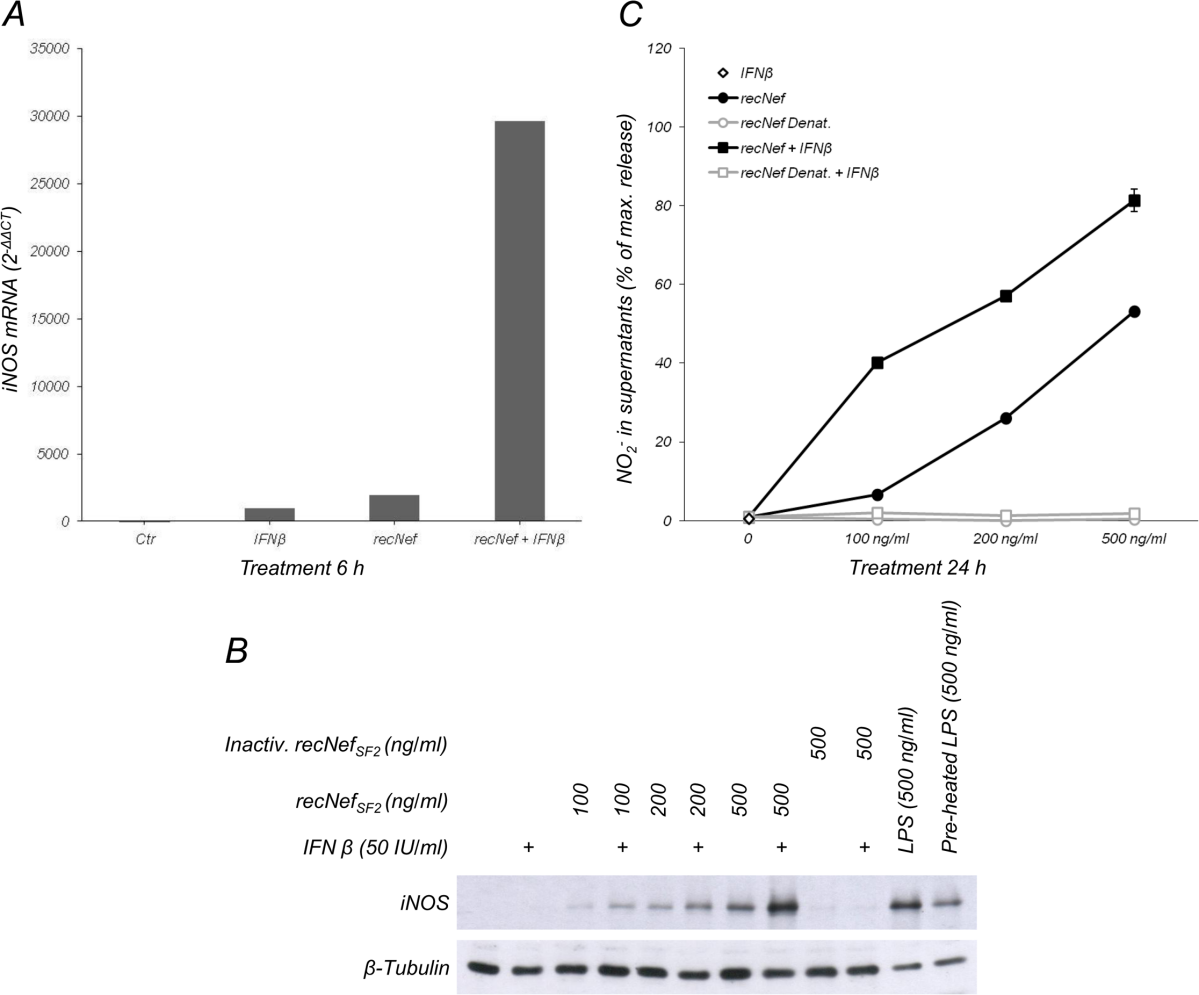
Nef synergizes with IFNβ in iNOS production. (**A**) BV-2 cells were treated for 6 h with myr^+^Nef_SF2_ (100 ng/ml), IFNβ (200 IU/ml) or a combination of both. iNOS mRNA was measured by real time RT-PCR as reported in the materials and methods section. (**B**) Cells were incubated for 24 h with the indicated dose of myr^+^Nef_SF2_ with or without IFNβ (50 IU/ml). Cells were also incubated with heat-inactivated recNef_SF2_ (inactiv. recNef, 500 ng/ml) or, as control, with LPS or pre-heated LPS (500 ng/ml each). Total cellular lysates were analyzed by Western Blot for iNOS expression. β-Tubulin expression was used as an internal loading control. (**C**) Cells were treated like in (**B**) and NO_2_^-^ content in supernatants was measured using the Griess colorimetric assay. Empty diamond: IFNβ; filled circles: myr^+^Nef_SF2_; gray empty circles: heat-inactivated myr^+^Nef_SF2_; filled squares: recNef_SF2_ plus IFNβ; gray empty squares: heat-inactivated myr^+^Nef_SF2_ plus IFNβ.

It is well recognized that stimuli that induce IRF-1 expression do not induce *per se* neither iNOS expression nor NO_2_^-^ production, rather, they “prime” the cells to respond to NF-κB-activating stimuli, improving their effect on iNOS regulation. This is, paradigmatically, the case of LPS and IFNγ combined treatment [46–48]. Therefore, we sought to test whether IFNβ has a priming effect in promoting Nef-induced iNOS expression and function. The results shown in Fig 5 demonstrate that combined treatment induced iNOS mRNA expression (Fig 5A), iNOS protein levels (Fig 5B) and NO_2_^-^ production (Fig 5C) to a greater extent compared to what is observed in cells exposed to myr^+^Nef alone.

### Nef myristoylation and conserved acidic cluster are essential to induce iNOS

**Fig 6:**
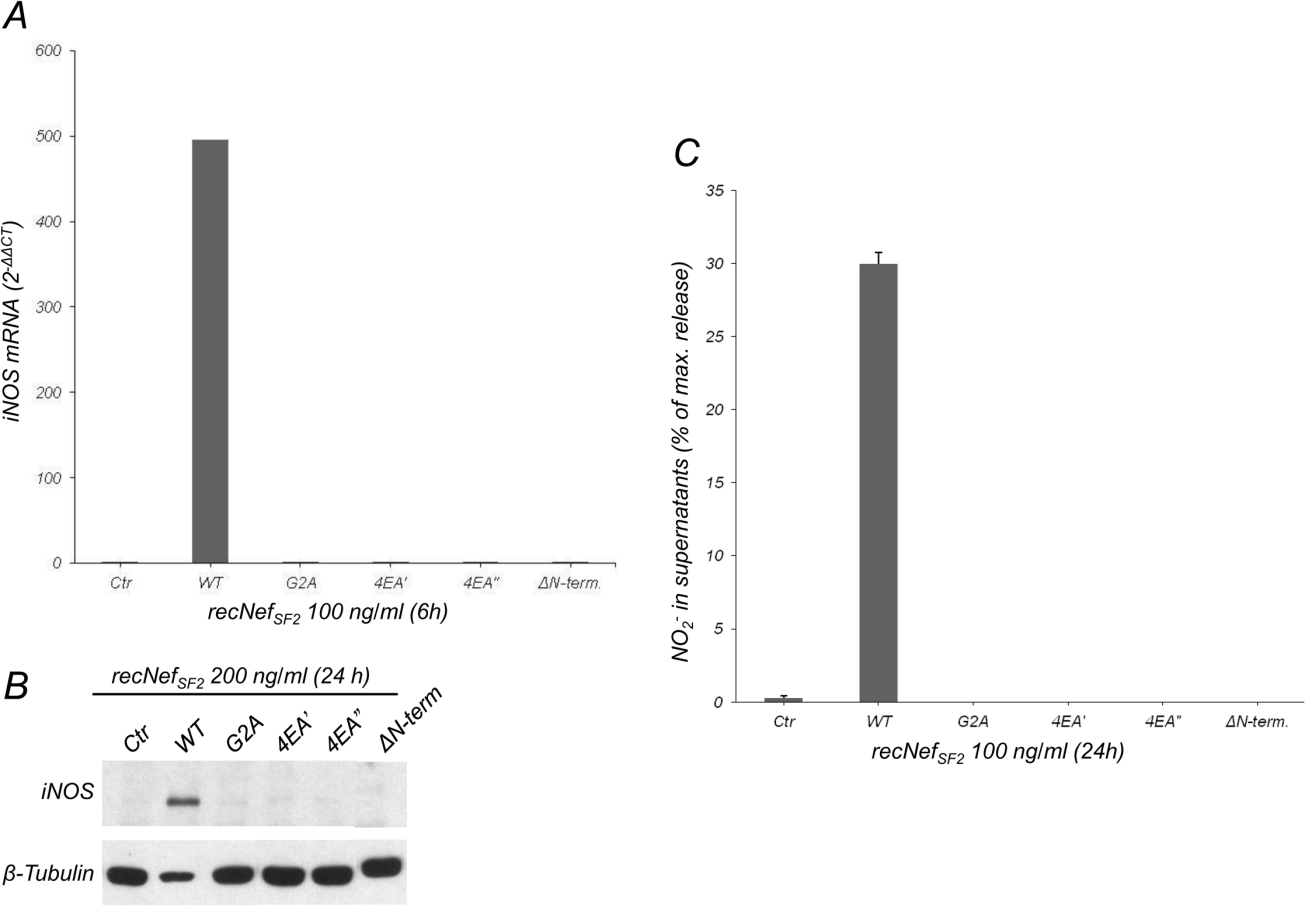
Nef myristoylation and the acidic cluster are essential to induce iNOS. BV-2 cells were left untreated or incubated with 100 ng/ml wild type (WT), G2A, two different preparations of the EEEE→AAAA mutant (4EA’ and 4EA”) and ΔN-terminal deleted myr^+^Nef_SF2_ proteins. (**A**) iNOS mRNA was measured by real time RT-PCR using total RNA isolated from cells treated for 6 h. (**B**) Total cellular extracts obtained from cells treated for 24h were analyzed by Western Blot using anti-iNOS antibodies, whereas β-tubulin expression levels were used as an internal loading control. (**C**) Supernatants collected from cells treated as in (**B**) were measured for NO_2_^-^ content using the Griess colorimetric assay. One out of three independent experiments is shown.

We previously demonstrated that Nef-mediated interference with cell signalling in human and murine macrophages treated with the viral protein required the integrity of both the N-terminal myristoylation site and the acidic cluster (AC) which consists of four glutamates [28, 32, 36]. To determine whether these motifs are also required for iNOS induction, BV-2 cells were treated with wild type myr^+^Nef_SF2_, a mutant in the myristoylation site (*i*.*e*. G2A), two different preparations of a myristoylation positive, AC-mutated viral protein (4EA’ and 4EA”), and a Nef-deletion mutant lacking the N-terminal anchor domain (∆N-term). The results shown in Fig 6 indicate that all the mutants tested failed to induce iNOS mRNA expression (Fig 6A), iNOS protein (Fig 6B), and NO_2_^-^ production alone or in IFNβ combined treatments (Fig 6C). Conversely, other Nef mutants we tested retained their pro-inflammatory capabilities and therefore their ability to induce iNOS expression and RNS production alone or in combined treatment with IFNβ. We tested Nef mutants affecting functional motifs involved in the interaction and down-regulation of CD4 (CAWL), the interaction with SH3-containing proteins (PxxPxP) or with proteins involved in endocytic pathways such as the V1H subunit of the vacuolar membrane ATPase or the adaptor protein complexes APs (DD and LL), as well as a loop mutant lacking the C-terminal flexible loop (data not shown).These results assign the myristoylation site and the AC as essential structural motifs for Nef-mediated iNOS regulation.

### Extracellular Nef induced the release of iNOS-dependent neurotoxic factors in murine microglial cells

**Fig 7:**
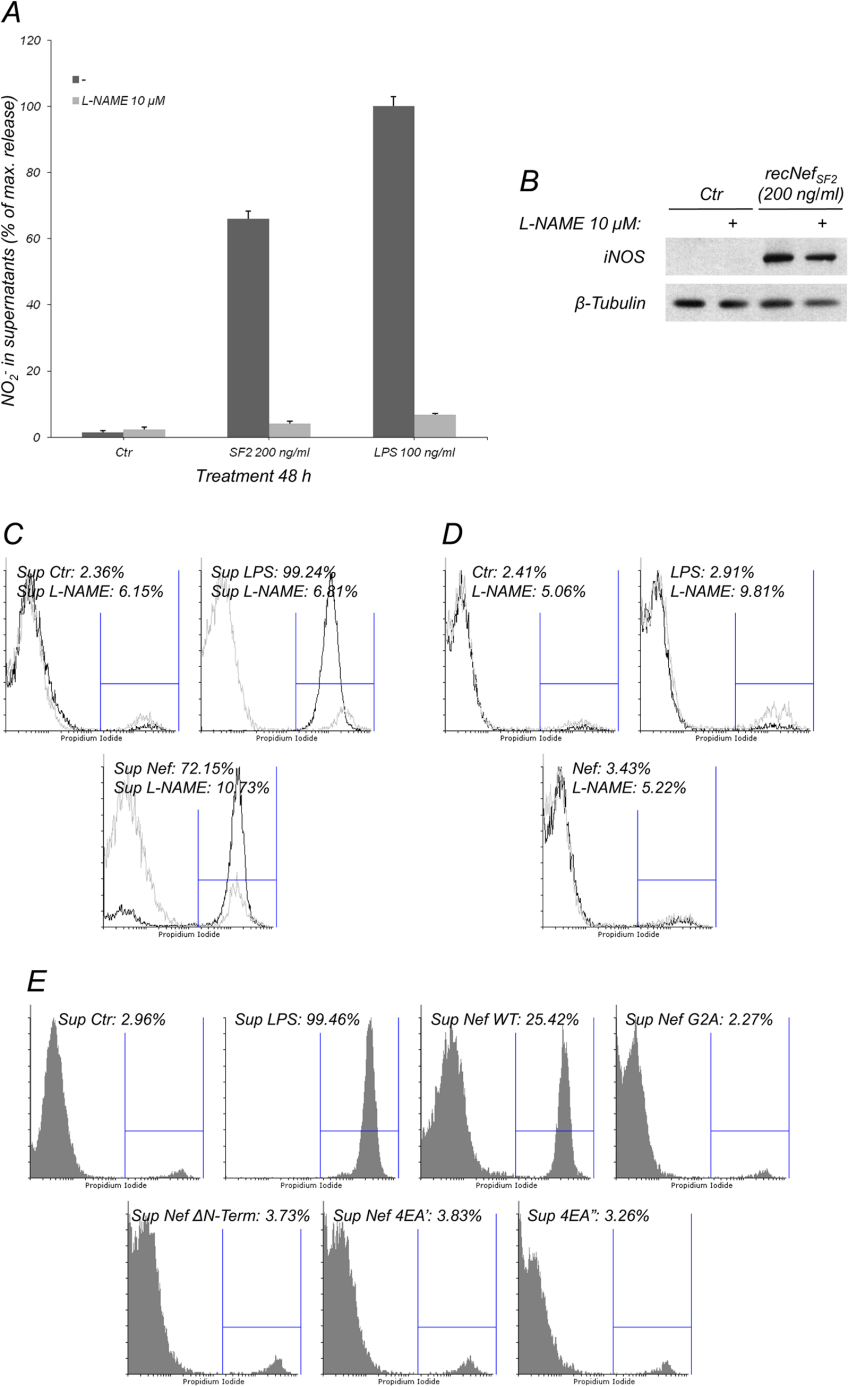
Nef-treated microglial cells release iNOS-dependent neurotoxic factors. (**A**) BV-2 cells were treated for 48 h with myr^+^Nef_SF2_ (200 ng/ml) in the presence (light gray bars) or absence (dark gray bars) of L-NAME at 10 μM concentration. Supernatants were collected and NO_2_^-^ content was measured using the Griess colorimetric assay. (**B**) iNOS expression was evaluated on total cell lysates by Western Blot using specific anti-iNOS antibodies (upper panel) and anti-β-tubulin antibodies as an internal loading control (lower panel). (**C**) NB41A3 cells were incubated for 24 h with supernatants from (**A**). (**D**) NB41A3 cells were treated for 24 h with myr^+^Nef_SF2_ (200 ng/ml) or with LPS (100 ng/ml). Black line: No L-Name treatment; Gray line: L-Name (10 μM). (**E**) NB41A3 cells were incubated for 24h with conditioned supernatants derived from untreated BV-2 cells or treated for 48 h with 200 ng/ml of wild type (WT), G2A, 4EA’, 4EA” and ΔN-terminal deleted myr^+^Nef_SF2_ proteins. In (**C)** to (**E)**, cell viability was measured by flow cytometry using Propidium Iodide (PI) dye exclusion and data were expressed as percentage of PI positive cells. One out of three independent experiments is shown.

It is thought that both viral products and cellular derived factors are mainly responsible for the neuronal loss observed in HAD [49]. To address whether Nef-dependent iNOS induction could have neurotoxic effects, the supernatant of BV-2 cells exposed to myr^+^Nef_SF2_ was collected and used to verify induction of cell death in NB41A3 murine neuroblastoma cell line. Supernatant collected from microglial cells treated for 48 h with myr^+^Nef, or with LPS used as positive control, induced cell death in NB41A3 cells (Fig 7C). This effect was completely abolished by the pre-treatment of BV-2 microglial cells with the NOS inhibitor L-NAME (Fig 7C), that prevented the Nef-dependent NO_2_^-^ production without affecting iNOS protein levels (Fig 7A and 7B). Treatment of NB41A3 cells for 24 h with myr^+^Nef, as well as LPS or L-NAME did not induce any cytotoxic effects (Fig 7D). Again, supernatants collected from BV-2 cells treated with G2A or 4EA mutants did not cause cell death in neuroblastoma cells (Fig 7E), demonstrating that the myr^+^Nef neurotoxic effects also require these domains. All together, these results show that Nef-stimulated microglial cells release NOS-dependent neurotoxic factor(s), whereas Nef itself is not neurotoxic.

## Discussion

Compelling evidence indicates that the neurocognitive disorders associated with the development and/or progression of AIDS are mainly due to the presence of infected macrophages/microglial cells characterized by a pro-inflammatory M1 profile, in the central nervous system (CNS). Indeed, neuroAIDS severity is directly correlated with the levels of pro-inflammatory cytokines and chemokines, whereas no direct correlation with the viral load has been demonstrated [50, 51]. Based on the theory of the “Trojan Horse” [52], HIV-1 is carried into the CNS by infected macrophages, the only immune cells able to cross the Blood Brain Barrier; the infection is then spread to other susceptible CNS-resident target cells, either competent (*i*.*e*. microglial cells) or defective (*i*.*e*. astrocytes) for HIV-1 replication [53]. The demonstrated presence of macrophage-tropic strains into the CNS further supports this hypothesis [54, 55]. Even if to the best of our knowledge, there is no evidence of Nef secretion from HIV-1 infected microglial cells and/or astrocytes, it has been reported that HIV-1 infected cells release Nef into the extracellular microenviromment. Nef was detected in the medium from the MOLT-4 lymphocyte cell line transfected with a Nef-producing baculovirus vector and it was quantified in 32 patient serum samples [56]. This evidence suggests that infected cells would release Nef through a non classical secretory pathway or after lysis. Then bystander cells like uninfected microglial cells in the case of CNS, might internalize Nef via endocytosis, pinocytosis or other unknow mechanisms, as we and others already reported for MDMs, dendritic and B cells [29, 57–61]. Once internalized, Nef could provoke the production of ROS and RNS as an integral part of a pro-inflammatory response [62, 63].

Our previous studies demonstrated that Nef treatment of human MDMs induces the pro-inflammatory response mainly through the activation of the NF-κB and IRF-3 transcription factors that lead to synthesis and release of IL-1β, IL-6, TNFα, CCL3/macrophage inflammatory protein 1a (MIP-1α), CCL4/MIP-1β and IFNβ [28, 29, 32]. Here we describe the ability of Nef to influence also the production of nitrogen reactive species through the induction of type 2 NOS, in an *in vitro* murine model of microglial cells exposed to the recombinant protein. The results reported in Fig 2 indicate a direct correlation between RNS production and Nef-induced upregulation of iNOS, both at the mRNA and protein level. As already reported for other canonical inducers, such as LPS and IFNγ, also in the case of Nef, iNOS induction is achieved through the activation of NF-κB and is reinforced by IRF-1 upregulation (Figs 3 and 4, respectively). Even if we cannot formally exclude the hypothesis that a surface receptor for Nef may activate a signalling cascade leading to iNOS induction, the observation that iNOS mRNA upregulation was inhibited by both cytochalasin D and 5-(N,N-dimethyl)-amyloride, two compounds which inhibit membrane ruffling and macropinocytosis [64, 65] argue against this hypothesis (data not shown).

In line with previous results, we show that iNOS induction and RNS production require the N-terminal myristoylation of Nef that influences Nef localization to the cellular membrane as well as the integrity of its conserved acidic cluster (Fig 6), which was shown to be pivotal for pro-inflammatory macrophage activation through the interaction with TNF receptor adapter factor 2 [28]. Interestingly, protein alignment studies on neurotropic strain-derived Nef from post-mortem biopsies revealed that some conserved amino acidic substitutions characterized these viruses compared to patient-matched blood-derived samples [66]. Two of them are 71R/K/T residue, in which the brain-derived viruses had arginine and lysine, whereas peripheral blood-derived HIVs had a mixture of threonine, lysine and arginine and 83G/A residue, in which brain-derived viruses displayed a glycine whereas blood-derived ones contained an alanine [66]. These residues are located immediately downstream of the Nef acidic cluster and they likely play a role in the stability and/or surface accessibility of this part of the protein that might also mediate the “pro-inflammatory potential” of the acidic cluster.

Studies performed to identify the phenotype of infected macrophages in the CNS revealed that both CD14^+^/CD45^+^ perivascular macrophages and multinucleated giant cells represent the major source of infected cells in macaques [67], whereas CD14^-^/CD45^-^ parenchymal microglial cells do not express any viral factor. From this point of view, the capacity of Nef to be transferred from infected cells to non-infected cells by exosomes and nanotubes or possibly via endocytosis as free protein (reviewed in [26]) should be emphasized. If this route of transmission is active also in the CNS, Nef-dependent iNOS induction and production of RNS might be achieved in non-infected microglial cells as well. As a consequence and regardless of the nature of infected cells in the CNS, a deregulated proinflammatory response could be induced, thereby promoting and/or accelerating the clinical onset of neurological disease [68]. Indeed, the production of matrix metalloproteinases MMP-2, -7, and -9, as well as IL-1, IL-6, TNFα, nitric oxide (NO), L-cysteine, and Ntox further disseminate immune activation in non-infected phagocytic cells.

Our results suggest a role of Nef in this macrophage- and/or microglial-based proinflammatory environment, possibly contributing to the induction of neuronal death and neuronal damage observed in the brain of AIDS patients [69]. Results obtained using the NB41A3 neuroblastoma cell line (Fig 7) demonstrated that the secreted factors induced by Nef in BV-2 cells, rather than Nef itself, are responsible for neuronal death assessed by PI uptake. These results are in conflict with reports indicating a direct neurotoxic activity of Nef due to its similarity to scorpion peptides [70]. L-NAME-dependent inhibition of neuronal death suggests that this phenomenon is dependent on NOS activity and NO production. Interestingly, NO_2_^-^_,_ provided to NB41A3 cells as NaNO_2_, was not toxic *per se* and supernatants collected from microglia treated with myr^+^Nef for only 24 h were unable to induce NB41A3 cell death (data not shown). The explanation of this observation is unknown at present, as the NO_2_^-^ content in supernatants collected from BV-2 cells treated for 24 and 48 h with Nef did not differ significantly (data not shown). It is conceivable that longer incubation times are needed to allow the release of other products that in concert with NO_2_^-^ induce cell death in NB41A3 cells. In this regard, it is interesting to note that both human and murine Nef-expressing astrocytes release CXCL10/IP-10, a chemokine shown to induce neuronal death, and that its neurotoxicity is more pronounced at 48 h than at 24 h after infection [71]. Further studies will be needed to evaluate iNOS induction and production of neurotoxic factor in a bona-fide human microglial cell system upon recombinant Nef treatment and/or Nef endogenous expression.

## Materials and Methods

### Cells cultures, recombinant Nef preparations and reagents

BV-2 cells, derived from primary murine microglial cells immortalized by transduction with v-*raf* and v-*myc* expressing J2 retrovirus, were characterized before [72]. BV-2 cells were cultured in Dulbecco’s modified essential medium (DMEM, Lonza, Milan, Italy) supplemented with 10% heat inactivated Fetal Calf Serum (iFCS, Lonza). NB41A3 neuroblastoma cell line [73, 74] was a kind gift from Ada Maria Tata, Department of Cellular and Developmental Biology, Sapienza University of Rome, Italy and was cultured in DMEM plus 10% iFCS. Primary mouse microglia were purified as previously described [75]. In brief, mixed glial cultures were established from the forebrain of 1-day old newborn CD1-Swiss mice. After 10 days *in vitro* microglial cells were detached from the astroglial monolayer by gentle manual shaking of the culture flasks; the supernatants were collected and centrifuged, and the cells were then reseeded on plastic surfaces, at the density of 10^5^ cells/cm^2^. After 1 h, the medium was replaced to remove non-adherent cells and microglial cells were allowed to grow for additional 24 h before starting experiments. The experimental procedures related to the use of newborn CD1 Swiss mice for establishment of primary microglial cultures have been reviewed and approved by the Italian Ministry of Health and the "Istituto Superiore di Sanitá" according to the italian law (Article 7, Legislative Decree 116/92) and Council Directive 86/609/EC. All surgery was performed under sodium pentobarbital anesthesia, and all efforts were made to minimize suffering.

Monocytes were purified as in [28]. No ethical approval from our and University “La Sapienza” ethics committees nor formal or verbal informed consent from blood donors were necessary to use buffy coats as sources of primary monocytes. Blood samples are collected for routine medical purpose and not specifically for this study. None of the authors collected the blood samples nor had any direct contact with the donors, or had access to any identifying information. M1 polarization was performed as in [76] by culturing monocytes for 6 days in RPMI, 10% FCSi supplemented with GM-CSF (Peprotech, Rocky Hill, NJ) at 50 ng/ml. According to [77], M1 phenotype was checked by flow cytometry using the following fluorchome-conjugated antibodies: CD14-FITC (clone UCHM1, Becton Dickinson, Research Triangle Park, NC), CD86-APC (clone IT2.2, BioLegend, San Diego, CA) and CD163-PE (clone GHI/61, BioLegend). At least ten thousand events were recorded using a FACs ARIA II sorter (Becton Dickinson) and the obtained data were analyzed using Flowing software (v.2.5.1, University of Turku, Finland).

Wild-type (WT) myristoylated Nef_SF2_, the mutant in the acidic cluster (E^66^EEE^69^→AAAA), the myristoylation deficient mutant (G2A) and the mutant lacking the first 44 amminoacids (ΔN-Term), were co-expressed with a N-myristoyl-transferase expression vector in *E*.*coli*, induced in presence of myristic acid and purified as C-terminal hexahistidine-tagged fusion proteins as previously described [78]. WT as well as mutants preparations of the viral protein were checked by SDS-PAGE and Coomassie staining after purification and titered by extinction coefficient measurement. Nef preparations were analyzed for the presence of endotoxin using the chromogenic Limulus amebocyte lysate endpoint assay QCL-1000 and, if required, purified using the EndoTrap endotoxin removal system (both from Lonza). Escherichia coli 0111:B4 lipopolysaccharide (LPS), used as positive control, and L-NAME were purchased from Sigma-Aldrich (Milan, Italy).

The highly specific IKKα/β inhibitor BMS-345541 [79, 80] was a kind gift from Dr. James R. Burke, Department of Immunology, Inflammation and Pulmonary Drug Discovery, Bristol-Myers Squibb Pharmaceutical Research Institute, Princeton, NJ.

Both murine IFNβ and anti-IFNβ NAb (neutralizing titer 4x10^6^ UI/ml) were a kind gift from Dr. Paola Borghi, Department of Cell Biology and Neurosciences, Istituto Superiore di Sanità, Rome, Italy.

### Western blot assay

Assays were performed as previously described [28, 32]. In particular, cells were washed twice with phosphate-buffered saline (PBS), pH 7.4, and lysed for 20 min on ice in Lysis Buffer: 20 mM HEPES, pH 7.9, 50 mM NaCl, 10 mM EDTA, 2 mM EGTA, 0.5% nonionic detergent IGEPAL CA-630 (Sigma-Aldrich), protease and phosphatase inhibitors (COMPLETE and phosphoSTOP, Roche, Milan, Italy). Whole-cell lysates were centrifuged at 6,000 g for 10 min at 4°C, and the supernatants were frozen at 80°C. Protein concentration of cell extracts was determined by the Lowry’s protein assay. Aliquots of cell extracts containing 20 to 50 μg of total proteins were resolved on 7% or 10% sodium dodecyl sulfate-polyacrylamide gel electrophoresis (SDS-PAGE) and transferred by electroblotting on nitrocellulose membranes (Sartorius, Gottingen, Germany) overnight at 30 V with a Bio-Rad Trans-Blot apparatus. For the immunoassays, membranes were blocked in 3% bovine serum albumin (BSA) fraction V (Sigma-Aldrich) in TTBS-EDTA (10 mM Tris, pH 7.4, 100 mM NaCl, 1 mM EDTA, 0.1% Tween 20) for 1 h at RT and then incubated overnight at +4°C with specific antibodies diluted in 1% BSA/TTBS-EDTA. Antibodies were used as follows: rabbit polyclonal antibodies anti-iNOS and anti-Iκ-B-α from Cell Signaling Technology (Beverly, MA); rabbit polyclonal antibodies anti-IRF-1 (C-20) and rabbit polyclonal anti-human iNOS (N-20) from Santa Cruz Biotechnology (Santa Cruz, CA); mouse monoclonal anti β-tubulin from ICN Biomedicals (Costa Mesa, CA). Immune complexes were detected with horseradish peroxidase conjugated goat anti-rabbit or goat anti-mouse antiserum (Calbiochem/Merck, Milan, Italy), followed by enhanced chemiluminescence reaction (ECL; Amersham Pharmacia Biotech, Milan, Italy). To reprobe membranes with antibodies having different specificities, nitrocellulose membranes were stripped for 5 min at RT with Restore Western Blot Stripping Buffer (Pierce, Rockford, IL) and then extensively washed with TTBS/EDTA.

### RNA isolation and real-time RT-PCR assay

Real-time reverse transcriptase-PCR assays were performed on total RNA as described elsewhere [28]. Briefly, RNA was isolated using the RNeasy mini kit (Qiagen, Milan, Italy) according to the manufacturer’s instructions. Five hundred nanograms of total RNA was reverse transcribed using oligo(dT)12-18 (Pharmacia-Biotech) as a primer and 50 units of Moloney murine leukemia virus reverse transcriptase enzyme (Gibco/Invitrogen, Milan, Italy). Quantitative real-time PCR was then performed on reverse-transcribed iNOS mRNAs using syber-green PCR master mix (Applied Biosysthems, Monza, Italy) according to manufacter’s instructions and the following primers: iNOS Forward: 5’- GGCAGCCTGTGAGACCTTTG-3’; iNOS Reverse: 5’-GCATTGGAAGTGAAGCGTTTC-3’. The expression of GAPDH (glyceraldehyde-3-phosphate dehydrogenase) was used to normalize the mRNAs levels (GAPDH Forward primer: 5’- TGAAGCAGGCATCTGAGGG-3’; GAPDH Reverse: 5’-CGAAGGTGGAAGAGTGGGAG-3’). Basal mRNA level observed in untreated cells was chosen as a calibrator.

### NO_2_^-^ production and quantification

NO_2_^-^ production was measured using the Griess colorimetric assay on supernatant of BV-2 microglial cells. Cells were treated in DMEM without red phenol plus 10% iFCS. Supernatants were collected, clarified by centrifugation (3,500 g, 5’, 4°C) and stored at -80°C. Seventy microlitres of supernatants were incubated in duplicate for 5’ at RT with 10 μl of 10 mM sulfanilamide, 10 μl of 10 mM HCl and 10 μl of 10 mM NEDA (N-1-napthylethylenediamine dihydrochloride, all from Sigma Aldrich). Samples absorbance at 550 nm was evaluated with a ELISA reader (EL800 Bio-Tek instruments, Inc., VT). Data were expressed as the percentage of maximum NO_2_^-^ release obtained by treating cells with LPS 100 ng/ml.

### Neuronal cell death assay

Supernatant collected from BV-2 cells treated with wt recNef (200 ng/ml) or with G2A and 4EA Nef mutants for 48 h in presence or not of L-NAME (10 μM) were centrifugated at 3,500 g, 5’, RT, filtered using a 0.22 μm filter and added to 50% confluent NB41A3 cells. Twenty four hours later cells were collected and analyzed evaluating by cytofluorimetry the percentage of propidium iodide (PI) positive cells. Ten thousand events were recorded using a DAKO Galaxy flow cytometer (Dako, Glostrup, Denmark). Data analysis was performed using Flowing software (v2.5.1, Turku Centre for Biotechnology, Finland).

## Supporting Information

S1 File Western Blot densitometric analysis of MDMs treated with Nef for 48 h. Panel of Fig 1F corresponding to human MDMs treated for 48 h with IFNγ, 100 ng/ml wild type myr^+^Nef_SF2_ plus IFNγ, heat pre-treated myr^+^Nef_SF2_ and IFNγ or 100 ng/ml LPS plus IFNγ was analyzed by densitometry using ImageJ software (v.1.48). Data were normalized using β-Tubulin expression and expressed as fold of induction using the value of untreated MDMs as reference.
(PDF)
