# Supplementary figures and images for: HIV-1 Myristoylated Nef Treatment of Murine Microglial Cells Activates Inducible Nitric Oxide Synthase, NO2 Production and Neurotoxic Activity
Source: PLoS One. 2015 Jun 11;10(6):e0130189. doi: 10.1371/journal.pone.0130189 (PMC4465743; doi:10.1371/journal.pone.0130189)

S1 File. Western Blot densitometric analysis of MDMs treated with Nef for 48 h.

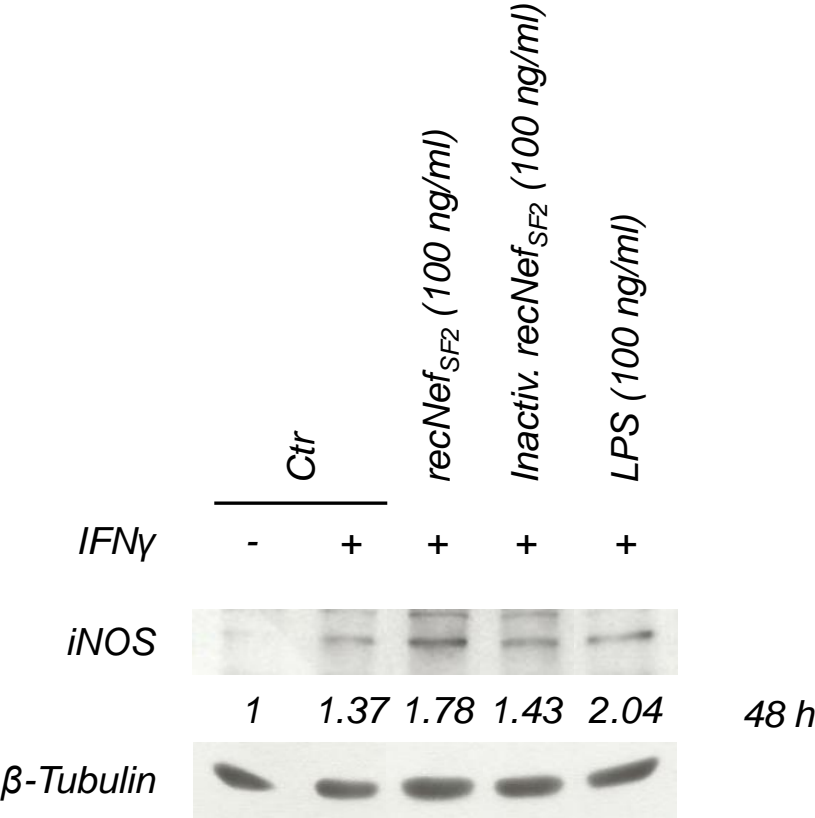

Supplement: S1 File — Panel of Fig 1F corresponding to human MDMs treated for 48 h with IFNγ, 100 ng/ml wild type myr+NefSF2 plus IFNγ, heat pre-treated myr+NefSF2 and IFNγ or 100 ng/ml LPS plus IFNγ was analyzed by densitometry using ImageJ software (v.1.48). Data were normalized using β-Tubulin expression and expressed as fold of induction using the value of untreated MDMs as reference. (PDF) [file pone.0130189.s001.pdf]
